# Impact of acute kidney injury in different ECMO modalities: a multicenter retrospective study on risk factors and mortality

**DOI:** 10.1080/0886022X.2026.2687905

**Published:** 2026-07-07

**Authors:** Yueguo Wang, Xin Wang, Xiancong Wang, Jian Sun, Yulan Wang, Xiongfeng Zhu, Huadong Meng, Shusheng Zhou, Kui Jin

**Affiliations:** a Department of Emergency Medicine, the First Affiliated Hospital of USTC, Division of Life Science and Medicine, University of Science and Technology of China, Hefei, China; b Graduate School of Bengbu Medical University, Bengbu, China; c Department of Emergency Medicine, The Third People’s Hospital of Hefei, Hefei, China; d Department of Emergency Medicine, the Third Affiliated Hospital of Anhui Medical University (Hefei First People’s Hospital), Hefei, China

**Keywords:** Extracorporeal membrane oxygenation, Acute kidney injury, risk factors, APACHE II score, intensive care unit

## Abstract

Acute kidney injury (AKI) is a common and serious complication in critically ill patients receiving extracorporeal membrane oxygenation (ECMO), significantly affecting mortality and long-term renal function. However, risk factors and clinical course of AKI across different ECMO modalities remain poorly understood. Herein, our study identified independent risk factors for AKI in ECMO patients and evaluated the effect of AKI severity on 30-day mortality. This multicenter retrospective cohort study enrolled patients from three ECMO centers (September 2019-June 2024). AKI was defined and staged according to KDIGO serum creatinine criteria within 7 days after ECMO initiation. Multivariate stepwise logistic regression identified predictors of moderate-to-severe AKI (stages 2–3). Cox proportional-hazards models assessed the association between AKI stage and 30-day mortality. Among 210 patients, 110 (52.4%) developed AKI stages 2–3 within 7 days. Serial monitoring showed a progressive increase in stage 2, while stage 3 plateaued. Moderate-to-severe AKI was independently associated with 30-day mortality. In the overall cohort, VA-ECMO modality and norepinephrine use were independent risk factors for AKI stages 2–3, while high fibrinogen (FIB) level and a history of cardiovascular disease (CVD) were protective. In the VV-ECMO subgroup, elevated lactate, bicarbonate, FIB, procalcitonin, and blood urea nitrogen levels, along with decreased total bilirubin and white blood cell counts were significantly associated with increased moderate-to-severe AKI risk. Herein, our study indicated that severe AKI independently predicts 30-day mortality in ECMO patients. VA-ECMO modality and NE use increase moderate-to-severe AKI risk, while high FIB level and CVD provide protection.

## Introduction

Acute kidney injury (AKI) is one of the most common and serious complications observed in patients undergoing ECMO therapy [1,2]. The pathophysiology involves multiple factors, including ECMO-associated hemodynamic alterations, systemic inflammatory activation, and the required anticoagulation regimens, all of which may contribute to kidney damage. Previous studies have reported that the incidence of AKI in ECMO patients ranges from approximately 50% to 70%, with differences noted across patient subgroups and ECMO modalities [3–5]. The development of AKI is strongly linked to increased hospital mortality and an elevated risk of progressing to chronic kidney disease (CKD) [6]. These consequences contribute to substantial social and economic burdens on both society and families [7].

Although AKI in ECMO patients has been widely investigated, existing evidence shows inconsistencies, and the effects of different AKI stages on patient outcomes are still not fully understood. In a single-center study by Kallur et al. [8], higher AKI stages were associated with increased likelihood of 30-day mortality, though these associations did not reach statistical significance in their cohort. Similarly, Chen et al. [9] found that among acute myocardial infarction patients complicated by cardiogenic shock supported by ECMO, severe AKI was associated with a markedly higher 1-year mortality (63.67% *vs*. 34.25%, all *p* < 0.001) and increased the risk of death approximately tenfold (HR 10.82, 95% *CI* 3.12–37.51, *p* < 0.001). Additionally, Bravi et al. [10] supported these findings, showing that AKI stage 3 significantly raised mortality risk in elderly patients with COVID-19 (OR = 2.02, 95% *CI* 1.04–3.9, *p* = 0.038). However, existing studies mainly focus on non-domestic populations and have limitations in their analysis of different ECMO modalities, including venoarterial ECMO (VA-ECMO) and venovenous ECMO (VV-ECMO). Furthermore, the long-term progression of AKI and its link to short-term mortality require further investigation.

Recent studies have provided additional insights into AKI in ECMO patients, particularly in those receiving VA-ECMO for postcardiotomy cardiogenic shock, as well as in related experimental models [11–14]. These studies demonstrate the high incidence and clinical impact of AKI and suggest that ECMO-associated AKI is a multifactorial complication involving hemodynamic instability, ischemia-reperfusion injury, and inflammatory responses, which warrants further research. In this context, our study aims to further explore the ECMO modality-specific risk factors and the early progression of AKI within 7 days.

To our knowledge, this retrospective study is one of the largest multicenter cohort studies in China to evaluate ECMO modality-specific risk factors and the early progression of AKI, alongside its relationship with patient mortality. The findings are intended to enhance early detection of high-risk patients, enable more precise clinical evaluation, and promote individualized treatment approaches, all of which contribute to improving the outcomes of ECMO patients. This manuscript has been previously published as a preprint on Research Square [15].

## Patients and methods

### Study design and participants

This multicenter retrospective cohort study was performed using the Chinese Emergency Triage Assessment and Treatment database (CETAT, version 2.0), which was developed by the Emergency Medical Specialist Alliance in China [16]. The study included patients who received ECMO therapy in the intensive care units (ICUs) of three tertiary hospitals: the First Affiliated Hospital of the University of Science and Technology of China (USTC), the Third Affiliated Hospital of Anhui Medical University (also known as Hefei First People’s Hospital), and Hefei Third People’s Hospital. Data was collected from September 2019 to June 2024.

Inclusion criteria were as follows: 1. Patients aged 18 years or older; 2. Patients who received ECMO therapy during the study period; 3. Availability of complete clinical data at the time of admission and prior to ECMO initiation. Exclusion criteria were as follows: 1. Patients who did not complete physician-prescribed treatments; 2. Incomplete or missing clinical data; 3. Survival durations of less than 48 h following ECMO initiation; 4. History of long-term hemodialysis or chronic renal failure; 5. Patients with advanced-stage cancer, pregnancy, or breastfeeding status; 6. Absence of essential laboratory or follow-up data.

### Definition and staging of AKI

Admission and reference Scr values were defined as previously described [17,18]. Admission Scr was defined as the first Scr measurement obtained during the hospitalization. When available, the baseline Scr was used as the reference value. In cases where baseline data were unavailable, the reference Scr was defined as the lowest of the admission Scr, the Scr measured within 24 h following ICU admission, or for patients without a history of CKD, the Scr estimated by back‑calculating the modification of diet in renal disease (MDRD) equation (Table S1) assuming a glomerular filtration rate of  75 mL/min/1.73 m^2^, in accordance with international guidelines [19–21]. AKI was defined and staged according to the 2012 Kidney Disease: Improving Global Outcomes (KDIGO) criteria, based on Scr levels within 7 days of ECMO initiation (Table 1). Once continuous renal replacement therapy (CRRT) was initiated, the patient was classified as having reached AKI stage 3. CRRT was initiated according to KDIGO recommendations. Indications included refractory pulmonary or peripheral edema unresponsive to diuretics, persistent hyperkalemia (>6.5 mmol·L^−1^), severe metabolic acidosis (pH < 7.1 or HCO_3_^–^ < 10 mmol·L^−1^), or marked azotemia [blood urea nitrogen (BUN) > 30 mmol·L^−1^] accompanied by uremic complications such as disturbance of consciousness, pericarditis, or bleeding diathesis. CRRT was also initiated in cases of anuria lasting ≥ 12 h or oliguria < 0.3 mL·kg^−1^·h^−1^ for ≥ 24 h.

**Table 1.** KDIGO Classification of renal function.

Stage	Scr Criteria
Stage 1	Increase in Scr to 1.5–1.9 times baseline or by ≥ 26.4 µmol/L within 48 h
Stage 2	Increase in Scr to 2.0–2.9 times baseline
Stage 3	Increase in Scr to 3.0 times baseline, or ≥ 352 µmol/L, or initiation of renal replacement therapy (RRT)

KDIGO, Kidney Disease: Improving Global Outcomes; RRT: Renal replacement therapy; Scr: Serum creatinine.

### Observational variables and grouping

Baseline clinical data were obtained from the electronic medical records, including demographic variables, Acute Physiology and Chronic Health Evaluation II (APACHE II) score, and history of chronic conditions. Additional parameters recorded at admission included vital signs, laboratory test results, indications for ECMO initiation [such as acute respiratory failure, cardiac arrest, and cardiogenic shock (non-cardiac arrest-related)], vasopressor use before ECMO initiation, and clinical outcomes. Patients were classified based on the highest stage of AKI observed during ECMO following KDIGO Scr criteria: no or mild AKI (stages 0–1), moderate AKI (stage 2), and severe AKI (stage 3). Furthermore, AKI stages 2–3 were combined into a single moderate-to-severe AKI group to facilitate the analysis of risk factors.

### Study endpoints

The primary endpoint of this study was the 30-day mortality rate. The second endpoint was the incidence of each AKI stage and its association with mortality risk. The date of death was defined as the time of the discharge event recorded as death in the CETAT database. Subgroup analyses were further conducted based on ECMO modality.

### Ethical approval and informed consent

This investigation retrospectively analyzed anonymized clinical data of adult patients receiving ECMO support. Written informed consent was waived by each participating hospital’s Ethics Committee, as the study involved no direct patient contact and posed no additional risk to participants. All research activities followed the Declaration of Helsinki and institutional ethical guidelines.

### Statistical methods

Statistical analysis was performed using Stata IC 16.0 and GraphPad Prism 10.0 software. Continuous variables were evaluated for normality using the Kolmogorov-Smirnov test. For data that were not normally distributed, values were reported as medians (interquartile range, IQR), and comparisons between groups were performed using the Kruskal-Wallis H test. Categorical variables are shown as counts (n) and percentages (%), and were analyzed using the chi-squared (χ^2^) test or Fisher’s exact test. A Cox proportional hazards model was used to evaluate the relationship between AKI stages and mortality risk, with AKI stages 0–1 as the reference group and adjusting for confounding variables. Hazard ratios (HR) and 95% confidence intervals (*CI*) were calculated. Kaplan-Meier survival curves and log-rank tests were used to compare 30-day mortality rates. Multivariate stepwise logistic regression analysis was applied to identify independent risk factors of moderate-to-severe AKI within 7 days using stages 0–1 as the reference group. The model’s discrimination was evaluated using receiver operating characteristic (ROC) curves. The variance inflation factor (VIF) test was used to assess multicollinearity among variables. Bootstrap resampling (1,000 samples) was used to validate the model. All statistical tests were two-tailed, with a *p* < 0.05 considered statistically significant.

## Results

### Baseline characteristics and dynamic evolution of AKI

**Figure 1.**
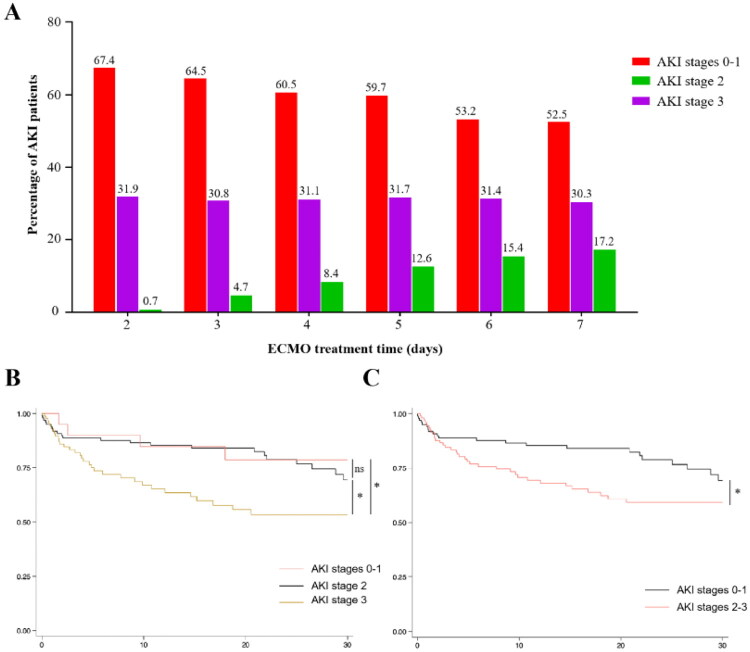
Dynamic evolution of AKI grading and 30-day survival analysis in patients receiving ECMO treatment. (A) Dynamic evolution of AKI grading within 7 days following ECMO initiation. (B) 30-day survival by individual AKI stage. (C) 30-day survival by combined AKI grade. AKI, acute kidney injury; ECMO, extracorporeal membrane oxygenation; ns, not significant. *p < 0.05.

A total of 210 patients receiving ECMO therapy were included in the study. Among them, 100 patients (47.6%) were classified into the AKI stages 0–1 group, while 110 patients (52.4%) were classified into the AKI stages 2–3 group. The incidence of AKI among VA-ECMO patients was 58.1%, and notably, over 75% of those with AKI stage 3 were supported with VA-ECMO. Comparisons of baseline characteristics, laboratory parameters, and clinical outcomes are provided in Tables 2–3. Compared with patients in the AKI stages 0–1 and stage 2 groups, those in the AKI stage 3 group were younger, had greater body weight, higher APACHE II scores, and a markedly higher prevalence of cardiac arrest and cardiogenic shock. Baseline characteristics of patients receiving VV-ECMO and VA-ECMO are presented in Table S2, and the distribution of CVD across ECMO modalities did not differ significantly within the cohort (Table S3). Patients with AKI stage 3 also demonstrated more frequent use of VA-ECMO and vasopressors, along with significantly elevated procalcitonin (PCT) and D-dimer levels and a marked reduction in platelet (PLT) count (*p* < 0.05). Dynamic monitoring of Scr within the first 7 days after ECMO initiation demonstrated a progressive decline in the proportion of patients in AKI stages 0–1, a gradual increase in those in stage 2, and a stable proportion of patients in stage 3 (Figure 1A). Kaplan-Meier survival analysis revealed that the 30-day cumulative survival rate was significantly lower in the AKI stage 3 group compared with the stages 0–1 and stage 2 groups (*p* < 0.05), while there was no significant difference between the AKI stages 0–1 and stage 2 groups (Figure 1B).

**Table 2.** Comparison of baseline characteristics by AKI stage.

Clinical Variables	AKI stages 0–1 (*N* = 100)	AKI stage 2 (*N* = 21)	AKI stage 3 (*N* = 89)	*p*-value
Demographics				
Male, *n* (%)	68 (68.0%)	18 (85.7%)	58 (65.2%)	0.19
Age, years	54 (39, 61)	53 (49, 61)	49 (33, 56)	0.02
Height, cm	168.0 (165.5, 170.0)	170.0 (168.0, 172.0)	168.0 (165.0, 172.0)	0.17
Weight, Kg	62.0 (60.0, 65.0)	58.0 (48.0, 64.0)	63.0 (62.0, 71.0)	0.01
Medical history				
Hypertension, *n* (%)	31 (31.0%)	9 (42.9%)	29 (32.6%)	0.57
Diabetes Mellitus, *n* (%)	17 (17.0%)	4 (19.0%)	13 (14.6%)	0.84
CVD, *n* (%)	17 (17.0%)	2 (9.5%)	9 (10.1%)	0.33
COPD, *n* (%)	6 (6.0%)	0 (0.0%)	4 (4.5%)	0.50
Vital signs at admission				
Temperature, °C	37.0 (36.0, 37.0)	37.0 (36.0, 37.0)	37.0 (36.0, 37.0)	0.05
Heart rate, bpm	99 (87, 112)	105 (99, 116)	108 (85, 122)	0.34
SBP, mmHg	114.0 (104.0, 127.5)	113.0 (107.0, 128.0)	113.0 (95.0, 129.0)	0.26
DBP, mmHg	75.0 (66.5, 84.5)	77.0 (67.0, 81.0)	67.5 (59.0, 78.0)	0.07
Disease severity				
APACHE II score	20.5 (17.0, 26.5)	20.5 (17.0, 20.5)	25.0 (20.5, 31.0)	<0.001
Indications for ECMO initiation				
Acute Respiratory Failure, *n* (%)	15 (15.0%)	1 (4.8%)	11 (12.4%)	0.42
Cardiac Arrest, *n* (%)	8 (8.0%)	0 (0.0%)	16 (18.0%)	0.02
Cardiogenic Shock, *n* (%)	14 (14.0%)	4 (19.0%)	35 (39.3%)	<0.001
CRRT use	0(0.0%)	0(0.0%)	84(94.4%)	<0.00
CRRT, *n* (%)				1
ECMO modality				
VA-ECMO, *n* (%)	42 (42.0%)	10 (47.6%)	70 (78.7%)	<0.001
Vasoactive drugs				
Norepinephrine, *n* (%)	74 (74.0%)	19 (90.5%)	87 (97.8%)	<0.001
Epinephrine, *n* (%)	72 (72.0%)	19 (90.5%)	85 (95.5%)	<0.001
Dopamine, *n* (%)	21 (21.0%)	5 (23.8%)	44 (49.4%)	<0.001

Data are presented as n (%) or median (interquartile range) as appropriate. *p* < 0.05 was considered statistically significant. ACS: Acute coronary syndrome; AKI: Acute kidney injury; APACHE II: Acute physiology and chronic health evaluation II; COPD: Chronic obstructive pulmonary disease; CRRT: continuous renal replacement therapy; CVD: Cardiovascular disease; ECMO: Extracorporeal membrane oxygenation; VA-ECMO: Venoarterial extracorporeal membrane oxygenation.

**Table 3.** Comparison of laboratory parameters and clinical outcomes.

Clinical variables	AKI stages 0-1 (*N* = 100)	AKI stage 2 (*N* = 21)	AKI stage 3 (*N* = 89)	*p*-value
Laboratory test results (Reference ranges)				
WBC, ×10⁹·L^–^¹, (3.5–9.5)	10.8 (7.7, 15.8)	9.5 (6.1, 15.4)	11.0 (6.9, 17.0)	0.61
Neu, ×10⁹·L^–^¹, (1.8–6.3)	8.7 (6.0, 13.0)	7.6 (4.5, 13.4)	9.2 (4.9, 15.2)	0.69
RBC, ×10¹²·L^–^¹, (3.8–5.1)	4.0 (3.6, 4.6)	4.4 (3.8, 4.8)	4.0 (3.4, 4.6)	0.22
Hb, g·L^–^¹, (115–150)	121.5 (109.0, 136.5)	131.0 (110.0, 139.0)	123.0 (102.0, 141.0)	0.56
PLT, ×10⁹·L^–^¹, (125–350)	175.5 (144.5, 236.0)	227.0 (160.0, 311.0)	145.0 (70.0, 206.0)	<0.001
CRP, mg·L^–^¹, (0–5)	34.4 (11.2, 95.5)	14.9 (5.6, 80.1)	58.7 (15.0, 121.4)	0.35
PCT, ng·mL^–^¹,(0–0.5)	0.3 (0.1, 1.1)	0.2 (0.1, 0.4)	1.1 (0.3, 2.9)	<0.001
Scr, μmol·L^–^¹, (46–92)	73.8 (52.4, 110.0)	55.8 (44.8, 91.7)	140.6 (74.5, 234.0)	<0.001
BUN, mmol·L^–^¹, (2.5–6.1)	7.2 (5.1, 9.6)	6.0 (4.6, 11.1)	9.2 (6.6, 13.4)	<0.001
TBIL, μmol·L^–^¹, (3–22)	14.7 (9.0, 22.3)	11.6 (6.1, 16.8)	17.0 (11.2, 30.3)	<0.001
DBIL, μmol·L^–^¹, (0–7)	6.5 (3.9, 8.3)	6.5 (3.9, 7.9)	7.8 (5.2, 14.7)	0.01
ALB, g·L^–^¹, (35–50)	32.9 (27.1, 36.8)	33.4 (25.6, 39.0)	28.9 (24.6, 34.9)	0.05
LAC, mmol·L^–^¹, (0.5–1.6)	2.9 (2.2, 5.2)	2.9 (1.9, 5.5)	5.5 (2.9, 6.8)	<0.001
APTT, s, (25–31.3)	30.4 (26.7, 38.2)	37.3 (28.5, 55.0)	42.7 (31.9, 62.1)	<0.001
PT, s, (8–14)	13.4 (12.5, 15.4)	14.4 (12.7, 15.9)	16.6 (14.0, 21.1)	<0.001
TT, s, (14–21)	17.5 (16.2, 19.6)	17.6 (16.7, 30.2)	20.4 (17.7, 37.6)	<0.001
D–dimer, μg·mL^–^¹, (0.01–0.55)	4.5 (1.1, 9.4)	4.6 (2.5, 6.8)	11.1 (4.1, 25.6)	<0.001
FIB, g·L^–^¹, (0.01–5)	4.0 (2.2, 5.8)	2.3 (2.0, 4.5)	2.9 (1.9, 5.2)	0.05
Arterial blood gas analysis (Reference ranges)				
PH, (7.35–7.45)	7.4 (7.3, 7.5)	7.4 (7.3, 7.4)	7.3 (7.2, 7.4)	<0.001
PCO_2_, mmHg, (35–45)	33.3 (29.8, 38.8)	34.0 (24.5, 53.0)	34.4 (31.1, 41.8)	0.64
PO_2_, mmHg, (80–100)	73.9 (54.3, 116.7)	73.9 (59.2, 214.0)	71.0 (48.0, 84.4)	0.17
HCO_3_^–^, mmol·L^–^¹, (21–28)	20.4 (19.9, 23.0)	19.9 (17.7, 24.0)	19.9 (16.8, 20.8)	<0.001
Clinical outcome				
Hospital length of stay, days	21 (11, 32)	28 (15, 46)	8.0 (3, 23)	<0.001
Death, *n* (%)	25 (25.0%)	5 (23.8%)	34 (38.2%)	0.11

Data are presented as *n* (%) or median (interquartile range) as appropriate. *p* < 0.05 was considered statistically significant. AKI: Acute kidney injury; APTT: Activated partial thromboplastin time; ALB: Albumin; BUN: Blood urea nitrogen; CRP: C-reactive protein; DBIL: Direct bilirubin; ECMO: Extracorporeal membrane oxygenation; FIB: Fibrinogen; Hb: Hemoglobin; HCO_3_^–^, Bicarbonate; LAC: Lactate; Neu: neutrophil; pH: Potential of hydrogen; PLT: Platelet; PCT: Procalcitonin; PCO_2_: Arterial partial pressure of carbon dioxide; PO_2_: Arterial partial pressure of oxygen; PT: Prothrombin time; RBC: Red blood cell; Scr: Serum creatinine; TBIL: Total bilirubin; TT: Thrombin time; WBC: White blood cell.

### Effect of AKI severity on 30-day mortality in ECMO patients

To evaluate the prognostic impact of AKI severity on 30-day mortality among ECMO patients, we adjusted for baseline disease severity using the APACHE II score as a covariate, consistent with previous studies [22,23]. Cox proportional regression analysis revealed that AKI stage 3 was significantly associated with an increased 30-day mortality, independent of APACHE II score adjustments (unadjusted HR = 2.01; 95% *CI* 1.19–3.40; *p* = 0.01; adjusted HR = 1.29; 95% *CI* 1.13–2.16, *p* = 0.04), identifying it as an independent risk factor for 30-day mortality. In subgroup analysis, AKI stage 3 remained a strong predictor of mortality in the VV-ECMO subgroup (unadjusted HR = 3.17, 95% *CI* 1.27–7.93, *p* = 0.01; adjusted HR = 2.09, 95% *CI* 1.29–5.50, *p* = 0.04), whereas no significant association was observed among patients receiving VA-ECMO (unadjusted *p* = 0.51; adjusted *p* = 0.44). Furthermore, each 1-point increase in APACHE II score corresponded to an approximate 10% −12% rise in mortality risk (HR = 1.10–1.12, *p* < 0.05) (Table 4). Consistently, Kaplan-Meier curves demonstrated a significantly lower 30-day survival probability for patients with moderate-to-severe AKI (stages 2–3) compared with those with stages 0–1 (*p* = 0.04) (Figure 1C).

**Table 4.** Cox Regression analysis of the association between AKI grade and 30-day mortality risk in ECMO patients.

	All ECMO (*N* = 210)			VA-ECMO (*N* = 122)			VV-ECMO (*N* = 88)		
Unadjusted APACHE II score	HR	95% *CI*	*p*-value	HR	95% *CI*	*p*-value	HR	95% *CI*	*p*-value
AKI stages 0-1	Reference								
AKI stage 2	0.80	0.31 – 2.10	0.66	0.82	0.23 – 2.89	0.76	0.66	0.15 – 2.96	0.59
AKI stage 3	2.01	1.19 – 3.40	0.01	1.25	0.64 – 2.46	0.51	3.17	1.27 – 7.93	0.01
AKI stages 2–3	1.67	1.01 – 2.77	0.04	1.19	1.03 – 2.23	0.03	1.78	1.20 – 4.14	0.04
Adjusted APACHE II score	HR	95% *CI*	*p*–value	HR	95% *CI*	*p*–value	HR	95% *CI*	*p*–value
AKI stages 0–1	Reference								
AKI stage 2	0.82	0.32 – 2.12	0.69	0.96	0.27 – 3.41	0.94	0.75	0.17 – 3.42	0.71
AKI stage 3	1.29	1.13 – 2.16	0.04	0.75	0.36 – 1.56	0.44	2.09	1.29 – 5.50	0.04
AKI stages 2–3	1.31	1.28 – 2.48	0.04	1.15	1.24 – 2.71	0.03	1.48	1.02 – 3.48	0.05
APACHE II score	1.10	1.07 – 1.13	<0.001	1.12	1.07 – 1.16	<0.001	1.10	1.03 – 1.18	<0.001

*p* < 0.05 was considered statistically significant.

### Modality-specific risk factors for moderate-to-severe AKI in ECMO patients

**Figure 2.**
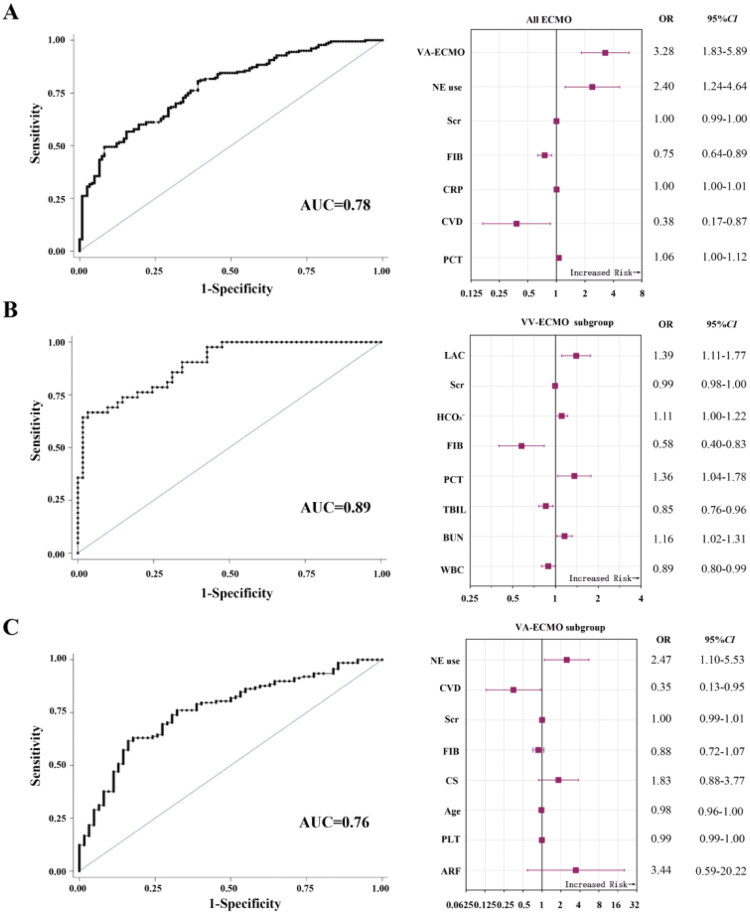
Multivariate logistic analysis of risk factors for moderate-to-severe AKI in ECMO patients. ARF, Acute respiratory failure; BUN, Blood urea nitrogen; CRP, C-reactive protein; CS, Cardiogenic shock; CVD, Cardiovascular disease; DBIL, Direct bilirubin; ECMO, Extracorporeal membrane oxygenation; FIB, Fibrinogen; HCO_3_^-^, Bicarbonate; Hb, Hemoglobin; LAC, Lactate; NE, Norepinephrine; PCT, Procalcitonin; PO_2_, Arterial partial pressure of oxygen; PLT, Platelet; Scr, Serum creatinine; TBIL, Total bilirubin; Temp, Temperature; TT, Thrombin time; VA-ECMO, Venoarterial extracorporeal membrane oxygenation; VV-ECMO, Venovenous extracorporeal membrane oxygenation; WBC, White blood cell.

To further evaluate the effect of ECMO modality on the risk of moderate-to-severe AKI, multivariate stepwise logistic regression analysis was performed in the overall cohort. As shown in Figure 2, the following variables were identified as independent risk factors for moderate-to-severe AKI: VA-ECMO modality (OR = 3.28, 95% *CI*: 1.83–5.89, *p* < 0.01) and NE use (OR = 2.40, 95% *CI*: 1.24–4.64, *p* = 0.01), while high FIB (OR = 0.75, 95% *CI*: 0.64–0.89, *p* = 0.001) and a history of CVD (OR = 0.38, 95% *CI*: 0.17–0.87, *p* = 0.02) appear to be a protective factor. To assess the discriminatory ability of these variables during early ECMO support, receiver operating characteristic (ROC) analysis was conducted. The overall model achieved an AUC of 0.78, indicating good predictive performance.

When stratified by ECMO modality, risk factor patterns varied between subgroups. In the VV-ECMO subgroup, higher levels of LAC (OR = 1.39, 95% *CI*: 1.11–1.77), HCO_3_^-^ (OR = 1.11, 95% *CI*: 1.00–1.22), PCT (OR = 1.36, 95% *CI*: 1.04–1.78), and BUN (OR = 1.16, 95% *CI*: 1.02–1.31) were associated with an increased risk of moderate-to-severe AKI. On the other hand, higher level of FIB (OR = 0.58, 95% *CI*: 0.40–0.83) and lower levels of TBIL (OR = 0.85, 95% *CI*: 0.76–0.96) and white blood cell (WBC) count (OR = 0.89, 95% *CI*: 0.80–0.99) were linked to a reduced risk (*p* < 0.05). The ROC analysis showed strong predictive performance, with an AUC of 0.89. However, in the VA-ECMO subgroup, NE use was the only independent risk factor (OR = 2.47, 95% *CI*: 1.10–5.53, *p* = 0.03), while a history of CVD appeared protective (OR = 0.35, 95% *CI*: 0.13–0.95, *p* = 0.04). The model for this subgroup achieved an AUC of 0.76. Although conditions like cardiogenic shock and acute respiratory failure prior to ECMO showed higher odds ratios, these associations were not statistically significant (*p* > 0.05), likely due to the small sample size. Additionally, as shown in Tables S4-S6, collinearity diagnostics demonstrated that all variables had VIF values <  2.0, with a mean VIF of 1.23 for the overall model, 1.25 for the VV-ECMO model, and 1.15 for the VA‑ECMO model, indicating no substantial multicollinearity. The bootstrap estimates were consistent in direction with those from the original stepwise logistic models, although several predictors lost statistical significance after bootstrapping (Tables S7–S9). Taken together, these results confirm that the associations identified in our regression models were robust and not confounded by collinearity among key covariates such as NE use, ECMO modality, and cardiogenic shock.

## Discussion

This retrospective study examined the dynamic epidemiology, prognostic implications, and risk factors of AKI in patients supported by ECMO, with specific attention to how ECMO modality influences the progression to moderate-to-severe AKI. Our findings indicated that the incidence of moderate-to-severe AKI was 52.4%, with AKI stage 3 frequent among those receiving VA-ECMO. During the initial 7-day period following ECMO initiation, the proportion of patients with AKI stage 2 gradually increased, while stage 3 patients remained stable. Importantly, AKI stage 3 was independently associated with 30-day mortality. Furthermore, independent risk factors for moderate-to-severe AKI included VA-ECMO modality and NE use, whereas high FIB level and a history of CVD appeared to confer a protective effect.

In this study, 42.4% of AKI patients developed AKI stage 3, which is slightly lower than the 53.8% reported by Vinclair et al. [24]. This discrepancy probably stems from different study populations and AKI definitions. Vinclair et al. restricted enrollment to VA-ECMO cases, whereas half of our cohort received VA-ECMO; this is a scenario consistent with real-world emergency use for refractory circulatory failure. VA-ECMO patients are more susceptible to severe AKI due to hemodynamic instability and frequent vasoactive drug use [25,26], whereas VV-ECMO patients generally maintain relatively stable hemodynamics [27]. Moreover, the definition of AKI severity differed between studies. While Vinclair et al. utilized the highest KDIGO stage recorded at any time during ICU admission, our analysis was confined to the initial 7-day period following ECMO initiation. This earlier and narrower observation window may have excluded patients of later renal deterioration, which likely contributes to the lower incidence observed in our study. Earlier investigations employing a similar 7-day diagnostic window have reported AKI stage 3 rates in line with our findings [28,29]. Clinically, concentrating on this early phase is meaningful, as published evidence suggests that approximately 80% of AKI episodes occur within the first week of hospitalization [18–20]. In addition, we observed that while the proportion of patients with AKI stage 3 remained largely constant over time, patients with AKI stage 2 increased during the observation period. This pattern underscores the importance of intensifying monitoring and implementing proactive renal protective measures in patients who progress to AKI stage 2. For individuals requiring extended ECMO support, however, the risk of AKI progression after the first week needs validation in larger multicenter studies. After adjusting for APACHE II scores, AKI stage 3 maintained an independent association with elevated 30-day mortality, a finding consistent with previous multicenter studies [24,30]. Vinclair et al. similarly found that AKI stage 3 at VA-ECMO initiation was independently linked to 1-year mortality (OR = 10.20), while our study similarly found that each 1‑point increase in APACHE II score raised mortality risk by 10% −12%, aligning with prior evidence [3,31]. These results confirm that APACHE II remains a reliable prognostic tool in ECMO patients and highlight the importance of interpreting mortality risk in the context of overall disease severity. Importantly, the differential prognostic impact of AKI stage 3 across ECMO modalities may be explained by distinct pathophysiological contexts. In VV-ECMO patients, AKI stage 3 has been consistently associated with increased mortality [2,32,33], suggesting that AKI severity may reflect the overall physiological deterioration. This is supported by evidence showing that severe AKI is often linked to microcirculatory impairment [34] and hypoxemia [35]. In contrast, VA-ECMO is generally used in patients with cardiogenic shock or cardiac arrest, where outcomes are predominantly determined by the severity of circulatory failure and myocardial dysfunction. In this setting, the influence of AKI severity on prognosis may be attenuated. Collectively, these findings suggest that the prognostic value of AKI stage 3 is context-dependent and varies according to ECMO modality and underlying disease severity. However, the shorter hospital length of stay (LOS) for patients with AKI stage 3 likely reflects early mortality rather than improved renal recovery. This finding should be interpreted with caution, as shorter hospital LOS could be misinterpreted as a positive outcome when it actually indicates early mortality. Therefore, it is crucial to consider both renal function and the patient’s overall health when evaluating outcomes.

Then, our study also indicated that VA‑ECMO modality and NE use were independently associated with an increased risk of moderate‑to‑severe AKI. This is biologically reasonable since VA-ECMO is mainly used for patients with severe circulatory failure, where non-pulsatile flow, increased afterload, and abnormal hemodynamic conditions can worsen renal perfusion. The bootstrap results revealed that several predictors lost statistical significance, suggesting limited model robustness and highlighting the need for further validation in larger, more diverse cohorts. Additionally, mechanical factors in the ECMO circuit may contribute to further renal hypoperfusion [36,37]. In addition, mechanical shear stress and blood exposure to artificial surfaces within the VA‑ECMO circuit (e.g., the oxygenator) can promote hemolysis and microthrombus formation [38,39]. NE use consistently predicted severe AKI (OR 2.40–2.47), corroborating Huette et al. [40]. Those authors showed that α1-adrenergic vasoconstriction and elevated systemic vascular resistance promote renal ischemia [41]. Moreover, higher FIB level also appeared as a protective factor against moderate-to-severe AKI, possibly reflecting preserved coagulation stability and better systemic homeostasis during ECMO support. This finding is consistent with prior studies indicating that coagulation imbalance contributes to microcirculatory dysfunction and subsequent organ injury [1,42]. Of particular interest was the observation that a history of CVD appeared to confer a protective effect in both the overall cohort and VA-ECMO subgroup (OR: 0.38 and 0.35, respectively). Several physiological mechanisms may explain this apparent protective effect. Episodes of chronic ischemia prior to ECMO cannulation, common in these patients, may induce a state of remote ischemic preconditioning (RIPC). Through RIPC, brief cycles of ischemia and reperfusion can provide systemic protective effects that also encompass the kidneys [43–45]. Patients with a known history of CVD often receive more timely fluid management and left ventricular unloading strategies at ECMO initiation [46]. Since most CVD patients are managed with VA-ECMO, a modality that rapidly improves systemic perfusion and attenuates renal ischemia. While these mechanisms may offer a potential explanation for the observed renal protection, the relatively small sample size of this analysis calls for cautious interpretation. In summary, the observed protective effect of CVD in our study is speculative and should not be interpreted as a definitive causal relationship. Future studies will involve additional analyses, including sensitivity analyses and stratified analyses by CVD subtypes, to better explore this association. In contrast, in VV-ECMO patients, the main risk factors for AKI were lactic acidosis and inflammation. This likely reflects the typical clinical profile of severe acute respiratory failure in VV-ECMO patients [47], wherein persistent hypoxia, cytokine release, and lactate accumulation induce renal cortical ischemia and tubular damage [48]. Unlike VA-ECMO, where hemodynamic instability and circulatory failure are the important factors inducing AKI, the pathophysiology in VV-ECMO is primarily driven by microcirculatory dysfunction and inflammatory responses.

Our study is one of the largest multicenter retrospective analyses of ECMO-associated AKI in China. However, several limitations should be considered. First, even with a sizeable overall cohort, the number of ECMO cases remains small, primarily due to the high costs and technical complexity constraining enrollment. Second, although we adjusted for illness severity using the APACHE II score, residual confounding may persist due to variables not captured in our dataset. These include ECMO circuit parameters, management protocols, and anticoagulation strategies. Third, several core technical parameters, such as blood flow, sweep gas flow, oxygenator type, cannulation strategy, and anticoagulation targets, were inconsistently documented across centers and could not be analyzed. Inter-center variations in ECMO management and the thresholds for initiating CRRT may influence AKI incidence. Finally, the definition of AKI did not include urine output (UO) criteria, which resulted in the exclusion of oliguric or anuric renal failure patients who did not meet the Scr threshold, likely resulting in an underestimation of the true AKI incidence. In future research, we will consider Scr and UO criteria based on the KDIGO recommendations.

## Conclusions

In this study, we observed a very high incidence of AKI in ECMO patients, which significantly affects their clinical prognosis. The risk of AKI differs greatly between VA-ECMO and VV-ECMO modalities. These findings highlight the need to incorporate modality-specific risk assessment and timely preventive strategies into clinical practice. Adjusting treatment according to the ECMO modality and associated risk factors can improve patient outcomes.

## Supplementary Material

Supplemental Material

## Data Availability

The datasets generated and analyzed during the current study are not publicly available due to sensitive personal clinical data but are available from the corresponding author upon reasonable request.
