# Supplementary material for: Impact of acute kidney injury in different ECMO modalities: a multicenter retrospective study on risk factors and mortality
Source: Ren Fail. 2026 Jul 7;48(1):2687905. doi: 10.1080/0886022X.2026.2687905 (PMC13347836; doi:10.1080/0886022X.2026.2687905)
Supplement: Supplemental Material [file IRNF_A_2687905_SM6896.docx]

**Table S1.** Estimated baseline creatinine.

| **Age (years)** | **Black males (mg/dl [µmol/l])** | **Other males (mg/dl [µmol/l])** | **Black females (mg/dl [µmol/l])** | **Other females (mg/dl [µmol/l])** |
| --- | --- | --- | --- | --- |
| 20-24 | 1.5 (133) | 1.3 (115) | 1.2 (106) | 1.0 (88) |
| 25-29 | 1.5 (133) | 1.2 (106) | 1.1 (97) | 1.0 (88) |
| 30-39 | 1.4 (124) | 1.2 (106) | 1.1 (97) | 0.9 (80) |
| 40-54 | 1.3 (115) | 1.1 (97) | 1.0 (88) | 0.9 (80) |
| 55-65 | 1.3 (115) | 1.1 (97) | 1.0 (88) | 0.8 (71) |
| >65 | 1.2 (106) | 1.0 (88) | 0.9 (80) | 0.8 (71) |

Estimated glomerular filtration rate = 75 (ml/min per 1.73 m^2^) = 186 × (serum creatinine [Scr ]) - 1.154 × (age) - 0.203 × (0.742 if female) × (1.210 if black) = exp(5.228 - 1.154 × In [Scr ]) - 0.203 × In(age) - (0.299 if female) + (0.192 if black).

**Table S2.** Baseline characteristics of VV-ECMO and VA-ECMO.

| **Clinical Variables** | **VV-ECMO**  **（N=88）** | **VA-ECMO**  **（N=122）** | ***P*-value** |
| --- | --- | --- | --- |
| **Demographics** |  |  |  |
| Male, n (%) | 62 (70.5%) | 82 (67.2%) | 0.62 |
| Age, years | 55.5 (42.5, 61.0) | 50.0 (34.0, 58.0) | <0.001 |
| Height, cm | 168.0 (168.0, 170.0) | 168.0 (165.0, 172.0) | 0.88 |
| Weight, Kg | 62.0 (60.0, 65.0) | 62.0 (60.0, 70.0) | 0.19 |
| **Medical history** |  |  |  |
| Hypertension, n (%) | 30 (34.1%) | 39 (32.0%) | 0.75 |
| Diabetes Mellitus, n (%) | 17 (19.3%) | 17 (13.9%) | 0.30 |
| CVD, n (%) | 8 (9.1%) | 20 (16.4%) | 0.12 |
| COPD, n (%) | 5 (5.7%) | 5 (4.1%) | 0.59 |
| **Vital signs at admission** |  |  |  |
| Temperature, ℃ | 37.0 (37.0, 37.0) | 37.0 (36.0, 37.0) | <0.001 |
| Heart rate, bpm | 102.0 (87.5, 115.5) | 105.0 (85.0, 120.0) | 0.58 |
| SBP, mmHg | 121.0 (110.0, 138.5) | 112.0 (96.0, 123.0) | <0.001 |
| DBP, mmHg | 78.0 (65.0, 88.0) | 70.5 (59.0, 78.0) | <0.001 |
| **Disease severity** |  |  |  |
| APACHE II score | 20.5 (17.0, 25.0) | 22.5 (20.5, 28.0) | <0.001 |
| **ECMO modality** |  |  |  |
| VA-ECMO, n (%) | 0 (0.0%) | 122 (100.0%) | <0.001 |
| **Vasoactive drugs** |  |  |  |
| Norepinephrine, n (%) | 69 (78.4%) | 111 (91.0%) | 0.01 |
| Epinephrine, n (%) | 67 (76.1%) | 109 (89.3%) | 0.01 |
| Dopamine, n (%) | 11 (12.5%) | 59 (48.4%) | <0.001 |
| **Laboratory test results (Reference ranges)** | | | |
| WBC, ×10⁹·L^-^¹, (3.5-9.5) | 10.0 (6.8, 15.0) | 11.0 (7.6, 16.9) | 0.37 |
| Neu, ×10⁹·L^-^¹, (1.8-6.3) | 8.1 (5.8, 13.2) | 9.1 (5.5, 14.8) | 0.47 |
| RBC, ×10¹²·L^-^¹, (3.8-5.1) | 4.0 (3.5, 4.4) | 4.1 (3.6, 4.7) | 0.14 |
| Hb, g·L^-^¹,(115-150) | 120.0 (105.0, 133.0) | 123.0 (109.0, 142.0) | 0.09 |
| PLT, ×10⁹·L^-^¹,(125-350) | 171.0 (118.5, 249.0) | 170.5 (114.0, 220.0) | 0.32 |
| CRP, mg·L^-^¹,(0-5) | 60.2 (13.2, 153.1) | 32.0 (10.4, 76.6) | 0.03 |
| PCT, ng·mL^-^¹,(0-0.5) | 0.3 (0.1, 1.1) | 0.7 (0.3, 1.1) | 0.02 |
| Scr, μmol·L^-^¹, (46-92) | 70.9 (52.4, 111.5) | 109.9 (67.0, 176.2) | <0.001 |
| BUN, mmol·L^-^¹, (2.5-6.1) | 7.8 (5.9, 12.1) | 7.7 (5.5, 12.2) | 0.64 |
| TBIL, μmol·L^-^¹, (3-22) | 13.6 (7.6, 20.8) | 16.5 (11.0, 29.0) | <0.001 |
| DBIL, μmol·L^-^¹, (0-7) | 6.5 (3.9, 8.5) | 7.2 (4.5, 13.1) | 0.07 |
| ALB, g·L^-^¹, (35-50) | 31.2 (24.5, 36.4) | 32.5 (26.8, 36.9) | 0.29 |
| LAC, mmol·L^-^¹, (0.5-1.6) | 2.9 (1.8, 5.3) | 3.8 (2.9, 5.5) | <0.001 |
| APTT, s, (25-31.3) | 30.5 (26.7, 38.2) | 38.3 (29.9, 60.0) | <0.001 |
| PT, s, (8-14) | 13.3 (12.6, 15.4) | 15.2 (13.4, 19.4) | <0.001 |
| TT, s, (14-21) | 17.5 (15.9, 19.1) | 19.9 (17.2, 41.2) | <0.001 |
| D-dimer,μg·mL^-^¹, (0.01-0.55) | 5.1 (2.2, 12.9) | 6.2 (2.2, 17.9) | 0.27 |
| FIB, g·L^-^¹, (0.01-5) | 4.8 (2.2, 6.2) | 2.9 (1.9, 4.6) | <0.001 |
| **Arterial blood gas analysis (Reference ranges)** | | | |
| PH, (7.35-7.45) | 7.4 (7.3, 7.5) | 7.4 (7.2, 7.4) | 0.02 |
| PCO_2_, mmHg, (35-45) | 33.3 (29.6, 42.1) | 34.0 (31.0, 40.4) | 0.67 |
| PO_2_, mmHg, (80-100) | 66.8 (48.4, 97.8) | 73.9 (59.1, 108.0) | 0.26 |
| HCO_3_^-^, mmol·L^-^¹, (21-28) | 20.1 (19.0, 24.1) | 19.9 (17.4, 20.8) | 0.05 |
| **Clinical outcome** |  |  |  |
| Incidence of AKI stages 2-3, n (%) | 30(34.1%) | 80(65.6%) | <0.001 |
| Hospital length of stay, days | 22.4 (12.4, 34.8) | 11.7 (2.5, 25.0) | <0.001 |
| Death, n (%) | 22 (25.0%) | 42 (34.4%) | 0.14 |

Data are presented as n (%) or median (interquartile range) as appropriate. *P* < 0.05 was considered statistically significant. ACS, Acute coronary syndrome; AKI, Acute kidney injury; APTT, Activated partial thromboplastin time; APACHE II, Acute physiology and chronic health evaluation II; ALB, Albumin; BUN, Blood urea nitrogen; CRP, C-reactive protein; COPD, Chronic obstructive pulmonary disease; CVD, Cardiovascular disease; DBIL, Direct bilirubin; ECMO, Extracorporeal membrane oxygenation; FIB, Fibrinogen; Hb, Hemoglobin; HCO₃⁻, Bicarbonate; LAC, Lactate; Neu, Neutrophil; pH, Potential of hydrogen; PLT, Platelet; PCT, Procalcitonin; PCO₂, Arterial partial pressure of carbon dioxide; PO₂, Arterial partial pressure of oxygen; PT, Prothrombin time; RBC, Red blood cell; Scr, Serum creatinine; TBIL, Total bilirubin; TT, Thrombin time; VA-ECMO, Venoarterial extracorporeal membrane oxygenation; WBC, White blood cell.

**Table S3.** CVD in VV-ECMO and VA-ECMO.

| **CVD** | **VV‑ECMO (N=88)** | **VA‑ECMO (N=122)** | **All ECMO (N=210)** |
| --- | --- | --- | --- |
| No | 80 (90.9%) | 102 (83.6%) | 182 (86.7%) |
| Yes | 8 (9.1%) | 20 (16.4%) | 28 (13.3%) |

Pearson χ² = 2.36, *P* = 0.125. CVD, Cardiovascular disease; VA-ECMO, Venoarterial extracorporeal membrane oxygenation; VV‑ECMO, Venovenous extracorporeal membrane oxygenation.

**Table S4.** VIF for all data parameters.

| **Variable** | **VIF** | **1/VIF** |
| --- | --- | --- |
| CRP | 1.66 | 0.60 |
| FIB | 1.57 | 0.64 |
| Scr | 1.16 | 0.86 |
| VA-ECMO | 1.12 | 0.89 |
| NE use | 1.12 | 0.89 |
| PCT | 1.11 | 0.90 |
| COPD | 1.06 | 0.94 |
| Mean VIF | 1.23 |  |

COPD, Chronic obstructive pulmonary disease; CRP, C-reactive protein; FIB, Fibrinogen; NE, Norepinephrine; PCT, Procalcitonin; Scr, Serum creatinine; VA-ECMO, Venoarterial extracorporeal membrane oxygenation; VIF, Variance inflation factors.

**Table S5.** VIF for parameters of VA-ECMO.

| **Variable** | **VIF** | **1/VIF** |
| --- | --- | --- |
| Height | 1.25 | 0.80 |
| Weight | 1.23 | 0.82 |
| CRRT | 1.18 | 0.85 |
| NE use | 1.16 | 0.86 |
| CA | 1.11 | 0.90 |
| Age | 1.10 | 0.91 |
| CS | 1.06 | 0.94 |
| CVD | 1.05 | 0.95 |
| Mean VIF | 1.15 |  |

CA, Cardiac arrest; CS, Cardiogenic shock; CVD, Cardiovascular disease; CRRT, Continuous renal replacement therapy; NE, Norepinephrine; VIF, Variance inflation factors.

**Table S6.** VIF for parameters of VV-ECMO.

| **Variable** | **VIF** | **1/VIF** |
| --- | --- | --- |
| CRP | 1.79 | 0.56 |
| FIB | 1.60 | 0.63 |
| LAC | 1.54 | 0.65 |
| ALB | 1.22 | 0.82 |
| HCO_3_^-^ | 1.22 | 0.82 |
| Hb | 1.21 | 0.82 |
| DBIL | 1.17 | 0.86 |
| WBC | 1.12 | 0.89 |
| PCT | 1.08 | 0.93 |
| TT | 1.07 | 0.94 |
| BUN | 1.03 | 0.97 |
| Mean VIF | 1.25 |  |

ALB, Albumin; BUN, Blood urea nitrogen; CRP, C-reactive protein; DBIL, Direct bilirubin; FIB, Fibrinogen; Hb, Hemoglobin; HCO₃⁻, Bicarbonate; LAC, Lactate; PCT, Procalcitonin; TT, Thrombin time; VIF, Variance inflation factors; WBC, White blood cell.

**Table S7.** All data bootstrap test.

| **Observed** | **Bootstrap** |  | **Normal** | **based** |  |
| --- | --- | --- | --- | --- | --- |
| **AKI** | **exp(b)** | **Std. Err.** | **z** | **P>z** | **95% *CI*** |
| VA-ECMO | 3.06 | 0.99 | 3.47 | 0.00 | 1.63~5.77 |
| NE use | 2.38 | 0.86 | 2.39 | 0.02 | 1.17~4.83 |
| Scr | 1.00 | 0.00 | 1.71 | 0.09 | 1.00~1.01 |
| FIB | 0.76 | 0.08 | -2.75 | 0.01 | 0.62~0.92 |
| CRP | 1.01 | 0.00 | 1.79 | 0.07 | 1.00~1.01 |
| COPD | 0.50 | 0.74 | -0.47 | 0.64 | 0.03~8.98 |
| PCT | 1.06 | 0.11 | 0.57 | 0.57 | 0.86~1.31 |

CRP, C-reactive protein; COPD, Chronic obstructive pulmonary disease; FIB, Fibrinogen; NE, Norepinephrine; PCT, Procalcitonin; Scr, Serum creatinine.

**Table S8.** VA-ECMO bootstrap test.

| **Observed** | **Bootstrap** |  | **Normal** | **based** |  |
| --- | --- | --- | --- | --- | --- |
| **AKI** | **exp(b)** | **Std. Err.** | **z** | **P>z** | **95% *CI*** |
| NE use | 2.07 | 0.72 | 2.10 | 0.04 | 1.05~4.09 |
| CVD | 0.44 | 0.20 | -1.77 | 0.08 | 0.17~1.09 |
| Scr | 1.00 | 0.00 | 2.24 | 0.03 | 1.00~1.01 |
| FIB | 0.80 | 0.07 | -2.66 | 0.01 | 0.67~0.94 |
| PO_2_ | 1.00 | 0.00 | -1.26 | 0.21 | 1.00~1.00 |
| TT | 1.00 | 0.01 | 0.77 | 0.44 | 0.99~1.01 |
| CS | 1.92 | 0.69 | 1.82 | 0.07 | 0.95~3.87 |
| Age | 0.99 | 0.01 | -1.14 | 0.26 | 0.98~1.01 |
| Temp | 0.72 | 0.17 | -1.36 | 0.17 | 0.45~1.15 |
| PLT | 1.00 | 0.00 | -1.31 | 0.19 | 0.99~1.00 |
| ARF | 1.22 | 0.57 | 0.42 | 0.68 | 0.49~3.06 |

ARF, Acute respiratory failure; CS, Cardiogenic shock; CVD, Cardiovascular disease; FIB, Fibrinogen; NE, Norepinephrine; PO₂, Arterial partial pressure of oxygen; PLT, Platelet; Temp, Temperature; TT, Thrombin time.

**Table S9.** VV-ECMO bootstrap test.

| **Observed** | **Bootstrap** |  | **Normal** | **based** |  |
| --- | --- | --- | --- | --- | --- |
| **AKI** | **exp(b)** | **Std. Err.** | **z** | **P>z** | ***95% CI*** |
| LAC | 1.01 | 0.03 | 0.22 | 0.83 | 0.95~1.07 |
| ALB | 0.99 | 0.02 | -0.55 | 0.58 | 0.94~1.03 |
| HCO_3_^-^ | 0.98 | 0.03 | -0.76 | 0.45 | 0.93~1.03 |
| Hb | 1.00 | 0.01 | -0.67 | 0.51 | 0.99~1.01 |
| FIB | 0.74 | 0.08 | -2.87 | 0.00 | 0.60~0.91 |
| CRP | 1.00 | 0.00 | 1.24 | 0.22 | 1.00~1.01 |
| PCT | 1.06 | 0.11 | 0.60 | 0.55 | 0.87~1.30 |
| BUN | 1.03 | 0.03 | 1.01 | 0.31 | 0.97~1.09 |
| WBC | 0.99 | 0.02 | -0.22 | 0.83 | 0.95~1.04 |
| DBIL | 1.00 | 0.02 | 0.21 | 0.84 | 0.97~1.04 |
| TT | 1.01 | 0.01 | 1.03 | 0.30 | 0.99~1.02 |

ALB, Albumin; BUN, Blood urea nitrogen; CRP, C-reactive protein; DBIL, Direct bilirubin; FIB, Fibrinogen; Hb, Hemoglobin; HCO₃⁻, Bicarbonate; LAC, Lactate; PCT, Procalcitonin; TT, Thrombin time; WBC, White blood cell.
